# Long-term positive severe acute respiratory syndrome coronavirus 2 ribonucleic acid and therapeutic effect of antivirals in patients with coronavirus disease: Case reports

**DOI:** 10.1590/0037-8682-0372-2020

**Published:** 2020-07-20

**Authors:** Bo Wei, Xiaofeng Hang, Ying Xie, Yuanjing Zhang, Jianrong Wang, Xinghao Cao, Jinzi J. Wu, Junxue Wang

**Affiliations:** 1 Department of infectious diseases, Changzheng Hospital, Naval Medical University, China.; 2 Ascletis Bioscience Co., Ltd., Hangzhou 310051, Zhejiang Province, China.; 3 Ascletis Pharmaceuticals Co., Ltd., Shaoxing 310051, Zhejiang Province, China.

**Keywords:** COVID-19, Antivirals, Long-term positive SARS-CoV-2

## Abstract

Coronavirus disease (COVID-19), caused by the severe acute respiratory syndrome coronavirus 2 (SARS-CoV-2), has been declared a pandemic. We herein report four COVID-19 cases with long-term positive viral ribonucleic acid (RNA) for about 61 days. Despite treatment with recombinant human interferon, convalescent plasma from COVID-19 patients, arbidol, etc., nucleic acid results were still positive for SARS-CoV-2. After treatment with ritonavir-boosted danoprevir (DNVr, 100/100 mg, once daily), all four patients showed two to three consecutive negative SARS-CoV-2 RNA and were thus discharged from hospital. Therefore, DNVr may be a potentially effective antiviral for COVID-19 patients with long-term positive SARS-CoV-2 RNA.

## INTRODUCTION

The coronavirus disease (COVID-19) pandemic, caused by the severe acute respiratory syndrome coronavirus 2 (SARS-CoV-2), has become a global public health threat. As of April 24, 2020, the number of confirmed COVID-19 cases was about 80,000 in China and 2.59 million worldwide^1^. However, there is still no effective antiviral therapy, and the main treatment is only supportive. Several clinical trials are currently testing antivirals against SARS-CoV-2, which may yield highly effective treatments for COVID-19^2^. 

Since the pandemic began, many COVID-19 patients have been discharged from hospitals, with a median hospitalization period of 12 days^3^. However, some COVID-19 patients have been reported to have long-term positivity for SARS-CoV-2 ribonucleic acid (RNA). Herein, we present four COVID-19 cases who had positive nucleic acid results for about two months.

## CASE REPORTS

### Case 1

**FIGURE 1:**
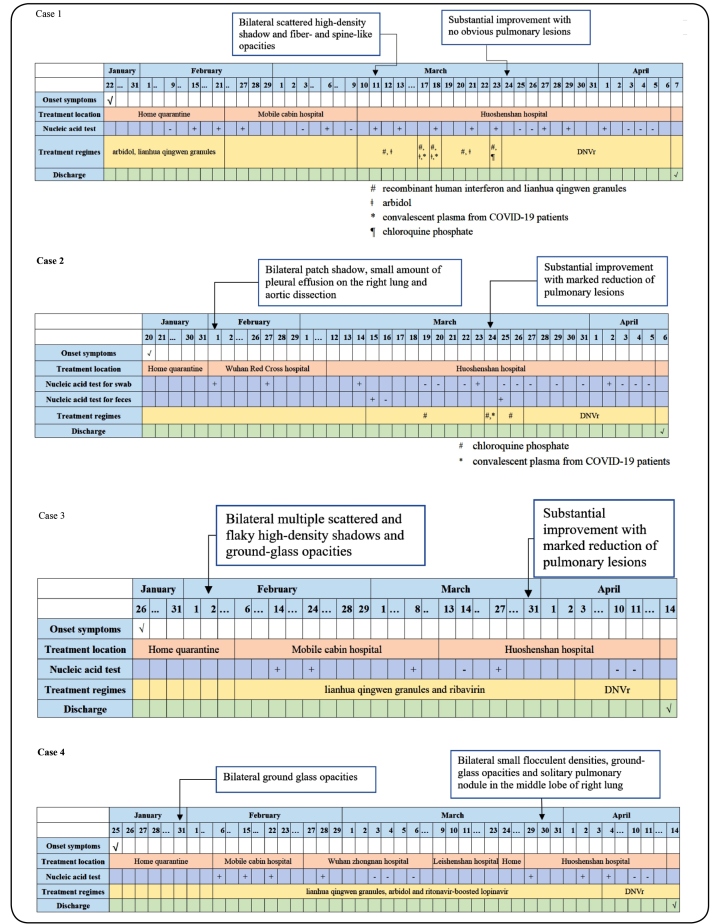
Clinical symptoms, serial viral test results of specimens, chest imaging, and treatment of COVID-19 patients. **DNVr:** ritonavir-boosted danoprevir, **CT:** computed tomography, **COVID-19:** coronavirus disease.

On March 10, 2020, a 50-year-old man, positive for SARS-CoV-2, was admitted to Wuhan Huoshenshan Hospital (Wuhan, China) after 47 days of cough and fever (Figure 1). On February 15, nucleic acid results for SARS-CoV-2 were positive. Previous treatments included arbidol (0.2 g, thrice daily) and lianhua qingwen granules (6 g, thrice daily). On admission, peripheral oxygen saturation was 96% in ambient air and respiratory rate was 18 breaths/minute. Immunoglobulin M (IgM) and Immunoglobulin G (IgG) levels for SARS-CoV-2 were, respectively, 53.12 g/L and 173.69 g/L (normal range for both: <10 g/L; Shenzhen YHLO Biotech Co., Ltd., Shenzhen, China). Computed tomography (CT) findings showed a bilateral, scattered, high-density shadow and fiber- and spine-like opacities. From March 10 to 22, convalescent plasma from COVID-19 patients (200 mL, March 17 and 18), recombinant human interferon (0.3 mL, atomized inhalation, once daily), lianhua qingwen granules (6 g, thrice daily), and arbidol (2 tablets, thrice daily) were administered as treatment; on March 23, chloroquine phosphate (2 tablets, twice daily) was administered instead of arbidol. On March 23, SARS-CoV-2 RNA were positive. A CT scan showed substantial improvement, with no obvious pulmonary lesions, on March 24. He was treated with ritonavir-boosted danoprevir (DNVr) alone (100/100 mg, one tablet twice daily). From April 3 to 5, nucleic acid test results for SARS-CoV-2 were all negative. SARS-CoV-2 IgM and IgG levels were markedly reduced (5.38 g/L and 108.68 g/L, respectively). On April 7, he was discharged from the hospital after three consecutively negative SARS-CoV-2 RNA. The duration from positive SARS-CoV-2 RNA to three consecutive negative results was 55 days.

### Case 2

On March 12, 2020, an 81-year-old man diagnosed with COVID-19 was admitted to Huoshenshan Hospital following 50 days of intermittent fever and 40 days of chest distress and shortness of breath (Figure 1). Type 2 diabetes mellitus, hypertension, and aortic dissection were associated comorbidities. On February 1, nucleic acid results for SARS-CoV-2 were positive. During prior hospitalization, digestive tract bleeding with black stool were observed; a CT scan showed bilateral patch shadow, a small amount of pleural effusion on the right lung, and aortic dissection. On admission, SARS-CoV-2 IgM and IgG levels were, respectively, 20.89 g/L and 187.92 g/L. Treatment included chloroquine phosphate (2 tablets, twice daily) and/or convalescent plasma from COVID-19 patients (400 mL) from March 15 to 26. On March 23, nucleic acid test results were positive for swab. On March 25, the patient’s feces were positive for SARS-CoV-2 RNA. A CT scan showed substantial improvement, with marked reduction of pulmonary lesions, on March 24. On March 27, he received DNVr treatment alone (100/100 mg, one tablet twice daily). On April 5, after three consecutive negative nucleic acid test results, he was discharged and transferred to another hospital for further treatment of comorbidities. The duration from positive SARS-CoV-2 RNA to three consecutive negative results was 65 days.

### Case 3

On March 13, 2020, a 42-year-old male with COVID-19 was admitted to Huoshenshan Hospital with 1 month of cough and fatigue and 10 days of fever (Figure 1). Prior history included a diagnosis of chronic hepatitis C in 2015 that was successfully cured, with sustained negative results of hepatitis C virus (HCV) RNA. On February 14, nucleic acid results for SARS-CoV-2 were positive. Previous treatments consisted of lianhua qingwen granules (6 g, thrice daily) and ribavirin (900-1200 mg, thrice daily). On admission, CT results showed bilateral, multiple, scattered, and flaky high-density shadows and ground-glass opacities. On March 27, nucleic acid test results were positive. On March 31, a CT scan showed substantial improvement, with marked reduction of pulmonary lesions. On April 3, he was treated with DNVr (100/100 mg, one tablet twice daily). On April 14, he was discharged from the hospital following two consecutive negative SARS-CoV-2 test results. The duration from positive SARS-CoV-2 RNA to two consecutive negative results was 58 days.

### Case 4

On March 29, 2020, a 33-year-old male with COVID-19 was admitted to Huoshenshan Hospital with 64 days of cough, chest distress, and shortness of breath as well as 7 days of backache (Figure 1). On January 31, CT results showed bilateral ground glass opacities. On February 6, nucleic acid results for SARS-CoV-2 were positive. On March 30, CT results showed bilateral small flocculent densities, ground glass opacities, and a solitary pulmonary nodule in the middle lobe of right lung. Previous treatments included lianhua qingwen granules (6 g, thrice daily), arbidol (0.2 g, thrice daily), and ritonavir-boosted lopinavir (2 tablets, once every 12 hours). On April 2, nucleic acid test results were positive. On April 4, he was treated with DNVr (100/100 mg, one tablet twice daily). On April 14, he was discharged from the hospital following two consecutive negative SARS-CoV-2 test results. The duration from positive SARS-CoV-2 RNA to two consecutive negative results was 66 days.

## DISCUSSION

The duration from positive SARS-CoV-2 RNA to consecutive negative results in the four cases presented ranged from 55 to 66 days (average: 61 days). All patients were treated with various antivirals, but all patients showed at least two consecutive negative nucleic acid results and were discharged from the hospital only after DNVr therapy. 

Long-term positivity of SARS-CoV-2 RNA may be due to the following reasons: First, immune system damage, immune tolerance, and escape. Inflammatory response plays an important role in the pathogenic effect of SARS-CoV-2, and severely maladjusted immune response could increase viral replication and induce tissue damage^4^. Postmortem examinations of six patients who died from COVID-19 showed that SARS-CoV-2 induced splenic nodule atrophy and reduced lymphocyte numbers^5^. Depletion of CD4+ T-cells is associated with delayed SARS-CoV-2 clearance from lungs^6^. Besides, the virus is capable of escaping innate immune responses, allowing for proliferation in the primarily infected tissues (airway epithelia)^7^. Second, the delayed viral clearance is possibly due to viral mutations. One specific viral isolate with a tri-nucleotide mutation was shown to lead to high viral loads, and the patient with this isolate was positive for SARS-CoV-2 for RNA about 45 days^8^. However, the mechanism behind the long-term positivity of SARS-CoV-2 needs further study. 

The false-negative rate has been estimated to be about 38% on the day of symptom onset and 66% on day 21^9^. Patients who recovered from COVID-19 did not show any respiratory symptoms (cough and expectoration), and imaging showed that lungs had returned to normal. The main reason for false-negative results may be due to sampling criteria. Guidelines from China and USA centers for disease control and prevention suggested that one of the criteria for hospital discharge is two consecutive negative nucleic acid results (sampling interval ≥24 hours) in respiratory specimens^10^ ^,^ ^11^. In the four cases presented, the nucleic acid results of SARS-CoV-2 using nasopharyngeal swabs were negative following treatment, but the results for sputum may be positive. Patients recovered from COVID-19 had long periods of positive SARS-CoV-2 RNA and specimens were from sputum swabbed from deep in the nasal passages^12^. Besides, the patients may “turn positive” in the future. Thus, long-term follow-up of patients is required to closely monitor the clinical symptoms and nucleic acid results of SARS-CoV-2.

At present, effective antivirals against SARS-CoV-2 have not been developed. However, a large number of medicines have been repurposed^2^, and clinical trials for these are ongoing, such as remdesivir (developed for SARS-CoV and Middle East Respiratory Syndrome-CoV) and danoprevir (inhibitor of HCV NS3/4A protease^13^, used in HCV treatment). All four cases presented in this report were treated with various medications, such as interferon atomization, arbidol, chloroquine, convalescent plasma from COVID-19 patients, and lianhua qingwen granules (all medicines recommended by the National Health Commission of the People’s Republic of China). After long-term treatment with these antivirals, SARS-CoV-2 RNA were still positive. Following treatment with DNVr, nucleic acid conversion was observed. Molecular docking-based virtual screening of all approved drugs showed that DNVr has the best binding energy to inhibit the main protease of SARS-CoV-2^14^. In a clinical trial of DNVr for COVID-19, all 11 patients were discharged from the hospital^15^. Thus, DNVr may be a potential antiviral for COVID-19 patients with long-term positive SARS-CoV-2 RNA. 

In conclusion, we presented four COVID-19 cases with long-term positive SARS-CoV-2 RNA (about 2 months) and DNVr may be effective in clearing SARS-CoV-2. However, a large-scale study investigating the efficacy of DNVr on SARS-CoV-2 is still needed. 
